# The E2F4 prognostic signature predicts pathological response to neoadjuvant chemotherapy in breast cancer patients

**DOI:** 10.1186/s12885-017-3297-2

**Published:** 2017-05-02

**Authors:** Kenneth M. K. Mark, Frederick S. Varn, Matthew H. Ung, Feng Qian, Chao Cheng

**Affiliations:** 1 Department of Molecular and Systems Biology, Geisel School of Medicine at Dartmouth, Hanover, NH 03755 USA; 2 Ministry of Education Key Laboratory of Contemporary Anthropology, School of Life Sciences, Fudan University, Shanghai, 200438 China; 3 Department of Biomedical Data Science, Geisel School of Medicine at Dartmouth, Lebanon, NH 03766 USA; 4 Norris Cotton Cancer Center, Lebanon, NH 03766 USA

**Keywords:** Breast cancer, Neoadjuvant chemotherapy, ChIP-seq, Transcription factor, E2F4, Pathologic complete response

## Abstract

**Background:**

Neoadjuvant chemotherapy is a key component of breast cancer treatment regimens and pathologic complete response to this therapy varies among patients. This is presumably due to differences in the molecular mechanisms that underlie each tumor’s disease pathology. Developing genomic clinical assays that accurately categorize responders from non-responders can provide patients with the most effective therapy for their individual disease.

**Methods:**

We applied our previously developed E2F4 genomic signature to predict neoadjuvant chemotherapy response in breast cancer. E2F4 individual regulatory activity scores were calculated for 1129 patient samples across 5 independent breast cancer neoadjuvant chemotherapy datasets. Accuracy of the E2F4 signature in predicting neoadjuvant chemotherapy response was compared to that of the Oncotype DX and MammaPrint predictive signatures.

**Results:**

In all datasets, E2F4 activity level was an accurate predictor of neoadjuvant chemotherapy response, with high E2F4 scores predictive of achieving pathologic complete response and low scores predictive of residual disease. These results remained significant even after stratifying patients by estrogen receptor (ER) status, tumor stage, and breast cancer molecular subtypes. Compared to the Oncotype DX and MammaPrint signatures, our E2F4 signature achieved similar performance in predicting neoadjuvant chemotherapy response, though all signatures performed better in ER+ tumors compared to ER- ones. The accuracy of our signature was reproducible across datasets and was maintained when refined from a 199-gene signature down to a clinic-friendly 33-gene panel.

**Conclusion:**

Overall, we show that our E2F4 signature is accurate in predicting patient response to neoadjuvant chemotherapy. As this signature is more refined and comparable in performance to other clinically available gene expression assays in the prediction of neoadjuvant chemotherapy response, it should be considered when evaluating potential treatment options.

**Electronic supplementary material:**

The online version of this article (doi:10.1186/s12885-017-3297-2) contains supplementary material, which is available to authorized users.

## Background

Neoadjuvant chemotherapy is a well-established treatment regimen used in managing patients with early-stage breast cancer [1]. In large or inoperable tumors, this therapy has been shown to substantially reduce tumor size allowing for easier removal and potentially breast conserving surgery [2, 3]. In some cases, administration of neoadjuvant chemotherapy may result in a substantial remission of the disease known as pathologic complete response (pCR), which is ascertained by pathological analysis of the resected tissue. However, in many cases, the disease may still be pathologically evident in the tissue, indicating the presence of residual disease (RD) [4]. Understanding the factors behind patients’ response to neoadjuvant chemotherapy may be beneficial in determining their personal treatment regimen and predicting their overall prognosis.

Though the benefits of neoadjuvant chemotherapy are clear, only a minority of breast cancer patients achieve pCR [5, 6]. The risk of RD means that neoadjuvant therapy may delay time to surgery without significant benefit [7]. Thus, it is important to better identify the patients most likely to achieve pCR. To date, prediction methods using imaging modalities such as mammography, radiology, and MRI have had limited success [8]. However, with the recent advent of high-throughput sequencing technology, several molecular assays have been developed to predict response to neoadjuvant chemotherapy [9–11]. One such assay, Oncotype DX [9] generates a predicted recurrence score based on the expression profile of 21 genes, and has shown promise in predicting neoadjuvant chemotherapy response in ER-positive patients. [12, 13] Another assay, Agendia’s MammaPrint [10, 11, 14] utilizes a 70-gene expression panel to determine a recurrence risk for early stage breast cancer. However, this assay must be combined with an additional 80-gene molecular subtyping assay, BluePrint [15], to predict neoadjuvant response [16].

We have previously developed a gene signature using chromatin immunoprecipitation sequencing (ChIP-seq)-inferred target genes of the transcription factor E2F4. E2F4 is a key regulator of the cell cycle, and patients exhibiting high expression of E2F4 target genes exhibit more severe cancer and shorter survival [17]. A follow-up study to our work revealed that the E2F4 signature is also predictive of neoadjuvant anthracycline-based chemotherapy response, even after adjusting for tumor grade [18]. In this study, we extend this work to assess the performance of our E2F4 signature in multiple independent datasets made up of diverse subtypes of breast cancer that undergo various regimens of neoadjuvant chemotherapy. We show that our signature performs comparably to the leading signatures on the market and demonstrate that a smaller gene signature composed of 28 E2F4 target genes and 5 control genes remains predictive of neoadjuvant chemotherapy response. Our results suggest that the transcriptional activity of E2F4 is predictive of chemotherapy response and demonstrates the potential of our E2F4 signature to be used as a clinical genomic assay to predict neoadjuvant chemotherapy.

## Methods

### Gene expression and clinical data

Breast cancer gene-expression datasets were downloaded from the NCBI’s Gene Expression Omnibus (GEO) database (GSE25066, GSE25055, GSE25065, GSE41998, GSE22093, GSE23988, GSE20271; Additional file 1), and together contained gene expression profiles for a total of 1129 primary patient tumors. An additional two-channel Agilent microarray breast cancer dataset was obtained from the Cancer Genome Atlas (Level 3) [19]. Each dataset chosen contained a minimum of 60 patients that underwent neoadjuvant therapy after tumor biopsy and included neoadjuvant therapy response information categorized as pCR or RD. For all datasets, processed data was used as available from GEO. For one-channel (Affymetrix) arrays, probesets were converted into gene symbol. In cases where multiple probesets existed for the same gene, the probeset with the highest average intensity across all samples was used.

### Calculation of the E2F4 signature

The 199-gene binary E2F4 target gene signature was determined as described previously [17]. This signature, along with a patient gene expression matrix were provided to the BASE (Binding Associated with Sorted Expression) algorithm [20, 21] to generate individual Regulatory Activity Scores (iRASs) representing E2F4 activity for each patient sample. For BASE to function, gene expression profiles from the input patient dataset must be quantile normalized and then, if the dataset is from a one-channel array, median centered. BASE then calculates the iRAS by ranking each patient’s normalized gene expression profile from high to low based on expression level and then determining the location of each E2F4 target gene in the ranked profile. Based on these ranked expression profiles, BASE then calculates two cumulative distribution functions comparing the relative expression of the E2F4 target genes (foreground function) to that of all other genes within the expression profile (background function). BASE calculates a preliminary E2F4 activity score by taking the maximal deviation between the two functions. Thus, a higher score indicates higher relative expression of the E2F4 target genes in the patient’s profile, meaning higher E2F4 activity, and a lower score indicates the opposite. Because this score is calculated as a difference between a foreground and background function, there will be no hard maximum or minimum and the scores instead will represent relative E2F4 activity level. BASE normalizes this score against the absolute value of the mean of a null distribution consisting of 1000 preliminary scores calculated from randomly permuted gene sets of equal size to the target gene set. The resulting final iRAS can be used to compare E2F4 activity between samples, with a higher iRAS indicating greater E2F4 activity compared to a lower iRAS.

### Survival analyses

A univariate Cox proportional hazards model was used to measure the association between patient E2F4 activity and survival outcome, while Kaplan-Meier curves were generated to visualize the survival distributions for all binary comparisons. *P*-values for the Cox models were determined using the Wald test and *p*-values for the Kaplan-Meier plots were calculated using the log-rank test. All survival analyses were performed in R through the *survival* package using the *coxph, survfit*, and *survdiff* functions for Cox proportional hazards models, Kaplan-Meier curves, and log-rank tests, respectively.

### Neoadjuvant response prediction

Samples were predicted as pCR or RD based on scores derived from the E2F4, Oncotype DX, or MammaPrint gene signatures. Oncotype DX and MammaPrint signature scores were calculated using the “oncotypedx” and “gene70” functions, respectively, from the *genefu* R package [22]. To predict neoadjuvant chemotherapy response for each prognostic signature, samples were ranked from low to high based on their signature-specific score. For each patient, a threshold was set, beginning with the lowest score, where all patients with a score less than or equal to the threshold were predicted to be RD and all samples above the threshold were predicted to be pCR. The sensitivity and specificity was then calculated for each threshold by comparing the predicted results to the actual results. Accuracy of each test was determined by calculating the area under the resulting receiver operating characteristics curve (AUC).

To test the performance of each prognostic signature in conjunction with clinical data, a Random Forest classifier was trained to predict pCR and RD status using the E2F4, Oncotype DX, and/or MammaPrint signatures as features, along with clinical data including age, tumor stage, tumor grade, estrogen receptor (ER) status, progesterone receptor (PR) status, HER2 status, and lymph node metastasis status. Random forest classification was performed in R through the *randomForest* package using the *randomForest* function under default settings. The performance of the model was evaluated by way of 10-fold cross validation where samples were randomly divided into 10 subsets, with 9 subsets used to train the model and predict the likely neoadjuvant response of the remaining validation subset. This process was repeated 10 times so that each sample was a part of the validation set at least once. Model effectiveness was assessed by calculating the AUC. This overall cross-validation procedure was repeated a total of 100 times to obtain an overall average AUC.

### Construction of the 33-gene E2F4 signature

A reduced E2F4 target gene signature of 34 genes was determined by identifying all E2F4 target genes whose own expression correlated highly (*R* > 0.8) with E2F4 scores in the TCGA BRCA dataset. Since all breast cancer datasets used in this study were obtained from one-channel array platforms, we used the Wang data (GSE2034) [23], which contains the expression profiles for 286 lymph-node-negative primary breast cancer patients, to define the formula for calculating E2F4 scores. First, we retrieved the log expression values of 28 genes from the dataset (of the initial 34 genes; 6 were missing in the Wang data) and normalized them into relative expression values by subtracting the average expression values (at log scale) of 5 control genes (*ACTB*, *GAPDH*, *RPLP0*, *GUSB*, *TFRC*). Second, we performed principle component analysis (PCA) on the normalized expression data for these 28 genes to obtain the first principle component (PC1). Since these genes are all highly correlated with E2F4 score across samples, PC1 explains a large fraction of their variation and is highly correlated with E2F4 score. Third, based on the PCA result, we calculated E2F4 using the following equation:

$$ E2 F4\  score={\beta}_1{e}_1+{\beta}_2{e}_2+{\dots +\beta}_n{e}_{n\ } $$

where *β*_*i*_ is the loading of gene *i* for PC1, *e*_*i*_ is the expression level of gene *i* in the sample, and *n* is the number of genes (*n* = 28). [24] Given this equation, E2F4 can be calculated when the relative expression levels (*e*_*i*_) of these 28 genes are quantified. The expression levels of these genes can be obtained by RT-PCR or other techniques using the same set of 5 control genes for normalization. In this analysis, we obtained their expression values from microarray data.

## Results

### E2F4 regulatory activity level predicts neoadjuvant response

**Fig. 1:**
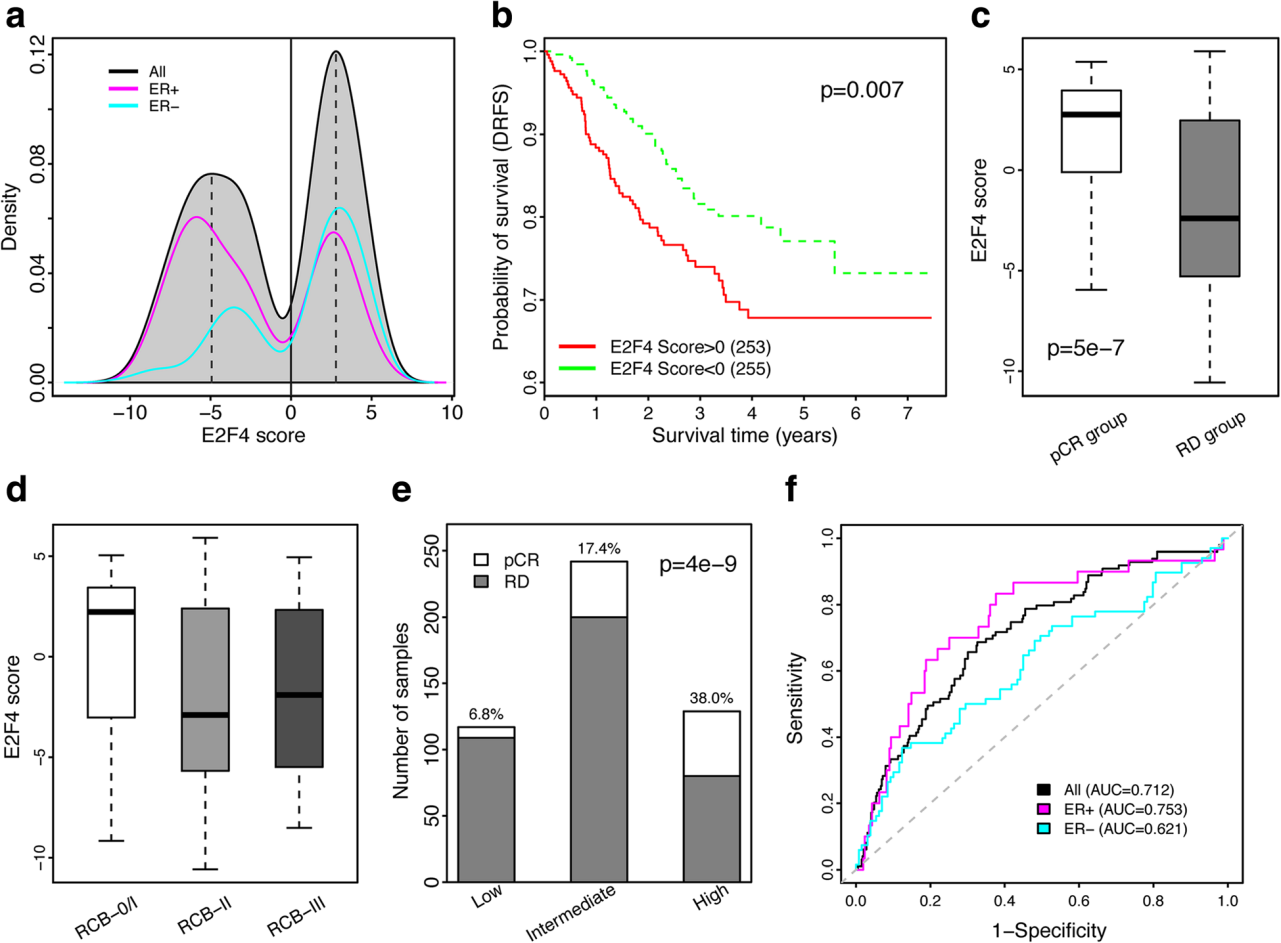
E2F4 activity and response to neoadjuvant chemotherapy. **a** Distribution of E2F4 activity scores for all patients (*grey*), ER-positive patients (*magenta*), and ER-negative patients (*aqua*). *Black dotted lines* indicate thresholds at which low, intermediate, and high E2F4 activity patient groups were stratified on. *Solid black line* indicates the threshold to stratify patients into low and high E2F4 activity groups for subsequent survival analyses. **b** Patients with high E2F4 activity (*red*) were associated with significantly shorter distant recurrence free survival time (DRFS) compared to patients with low E2F4 scores (*green*). Vertical hash marks indicate censored patients. **c** Comparison of E2F4 activity scores between patients achieving pathological complete response (pCR) and patients with residual disease (RD). **d** Comparison of E2F4 activity between patients with varying residual cancer burden: RCB-0/1 (*white*), RCB-II (*grey*) and RCB-III (*dark grey*) **e** Percentages of pCR and RD patients in E2F4 activity groups. **f** Receiver Operating Characteristic (ROC) curves for pCR prediction using E2F4 activity scores as features and 10-fold cross validation. ROC curves were generated for all (*black*), ER-positive only (*magenta*), and ER-negative only (*aqua*) patients

To examine the differences in E2F4 activity between pCR and RD patients, we calculated an E2F4 iRAS for each tumor in the Hatzis et al. dataset, which contains gene expression and clinical information for patients who underwent neoadjuvant chemotherapy [25]. Examining the scores across samples revealed that they were distributed in a bimodal fashion (Fig. 1a). Subsetting these scores by ER status revealed that each group roughly followed a bimodal distribution as well; though ER-negative patients tended to be enriched for high E2F4 iRASs, a likely reflection of their higher proliferation rates. To examine how E2F4 activity affected patient survival in this dataset, we stratified the patients into high (iRAS >0) and low (iRAS <0) E2F4 activity groups and compared their two survival distributions using a log-rank test (Fig. 1b). Patients with low E2F4 activity had significantly longer survival times than patients with high E2F4 activity (*p* = 7e-03; log-rank test), consistent with our previous findings [17]. The E2F4 score remained significant when used as a continuous variable in a univariate Cox proportional hazards model (*p* = 8e-3, HR = 1.09; Wald test).

We next examined the association between E2F4 score and neoadjuvant chemotherapy response. Patients that exhibited pCR had significantly higher E2F4 scores compared to RD patients (*p* = 5e-07, Wilcoxon rank-sum test; Fig. 1c), suggesting a potential role of E2F4 in response prediction. To further examine this relationship, we stratified patients by degree of residual cancer burden (RCB) as defined in the Hatzis dataset, with categories consisting of RCB-0 (pCR) to RCB-III (extensive RD). Patients with lower RCB tended to have higher E2F4 iRASs compared to higher RCB patients (Fig. 1d). Specifically, we found that patients with RCB-0 (pCR) or RCB-I (minimal RD) had significantly higher E2F4 iRASs than RCB-II (moderate RD) and RCB-III (extensive RD) patients (*p* = 3e-07 and 8e-05, respectively; Wilcoxon rank-sum test). Together, these results indicate that patients exhibiting high E2F4 activity were more likely to experience pCR.

To further validate the association we observed between E2F4 iRAS and neoadjuvant therapy response, we stratified patients into low, intermediate, and high E2F4 activity groups based on the distribution of E2F4 iRASs (dotted lines, Fig. 1a). Thresholds for each group were based off local maxima within the E2F4 score distribution, with the low class consisting of patients whose scores were less than the negative local maxima, the high class consisting of patients with scores greater than the positive local maxima, and the intermediate class consisting of the patients in between the high and low thresholds. Interestingly, we found that the class-specific pCR rates rose with each group from low to high, increasing from 6.8% to 17.4% to 38% (Fig. 1e). Furthermore, patients in the combined intermediate and high groups exhibited significantly higher rates of pCR compared to the low group (*p* = 4e-09; Fisher’s exact test). These results further suggested that E2F4 activity level can serve as a good predictor of pCR in breast cancer. To test this hypothesis, we used the E2F4 score of each patient as a threshold to classify patients as pCR or RD. This classification system achieved high accuracy, with an AUC of 0.71 (Fig. 1f). Stratifying samples into ER status and repeating this procedure resulted in AUCs of 0.75 and 0.62 for ER-positive and ER-negative, respectively. Together, these results indicate that the E2F4 iRAS by itself is a good predictor of pCR achievement after neoadjuvant chemotherapy.

**Fig. 2:**
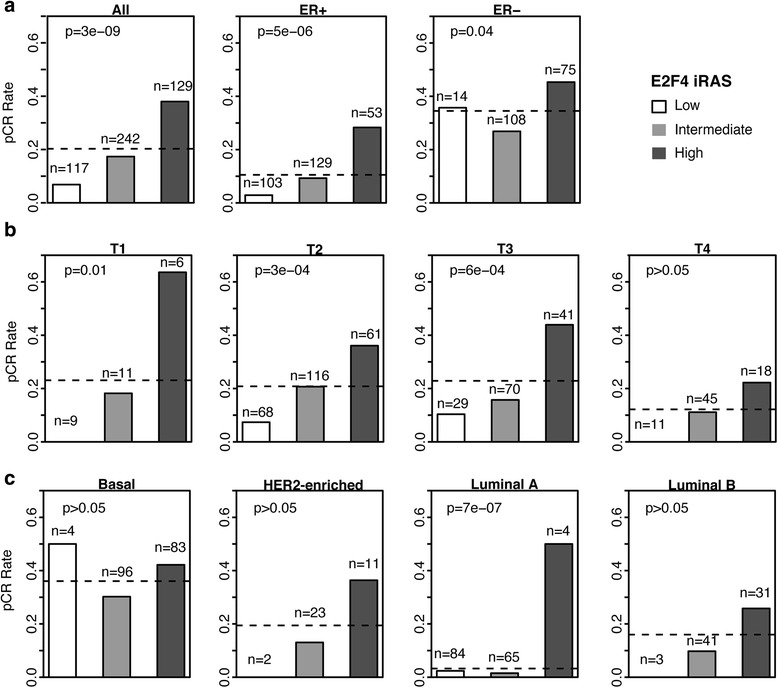
Percentage of patients achieving pCR in E2F4 activity groups after stratification on clinicopathological characteristics in the Hatzis dataset. **a** Percentage of patients achieving pCR with low (*white*), intermediate (*grey*), and high (*dark grey*) E2F4 activity groups for all, ER-positive, and ER-negative patients, respectively. **b** Percentage of patients achieving pCR with low (*white*), intermediate (*grey*), and high (*dark grey*) E2F4 activity groups in patients with different tumor stage. **c** Percentage of patients achieving pCR with low (*white*), intermediate (*grey*), and high (*dark grey*) E2F4 activity groups in patients belonging to different molecular subtypes. In all panels, horizontal dotted line indicates the percentage of pCR patients without stratifying based on E2F4 activity. *P*-values were calculated using the χ^2^ test

While our E2F4-based classification achieved good prediction accuracy across all samples, it may have been confounded by subtype-specific composition of the pCR and RD groups. To address this, we examined the association between E2F4 activity and neoadjuvant therapy response across different subgroupings of breast cancer, including ER status (Fig. 2a), tumor stage (Fig. 2b), and molecular subtype (Fig. 2c). For each subcategory, the rate of pCR was compared between the low, intermediate, and high E2F4 groups. In nearly all subcategories, the E2F4-high group exhibited the highest rate of pCR with chi-square tests indicating that there was a significant difference in pCR rate between the three groups. An exception to this trend was observed in subcategories known for more severe, highly proliferative cancers, such as the basal and ER negative subtypes and high stage tumors, where the differences in E2F4 iRAS were less pronounced. Based on these results, it is unlikely that E2F4-based classification was confounded by the composition of clinical features in the pCR and RD groups.

### Comparison of the E2F4 signature with other clinically-available prognostic assays

**Fig. 3:**
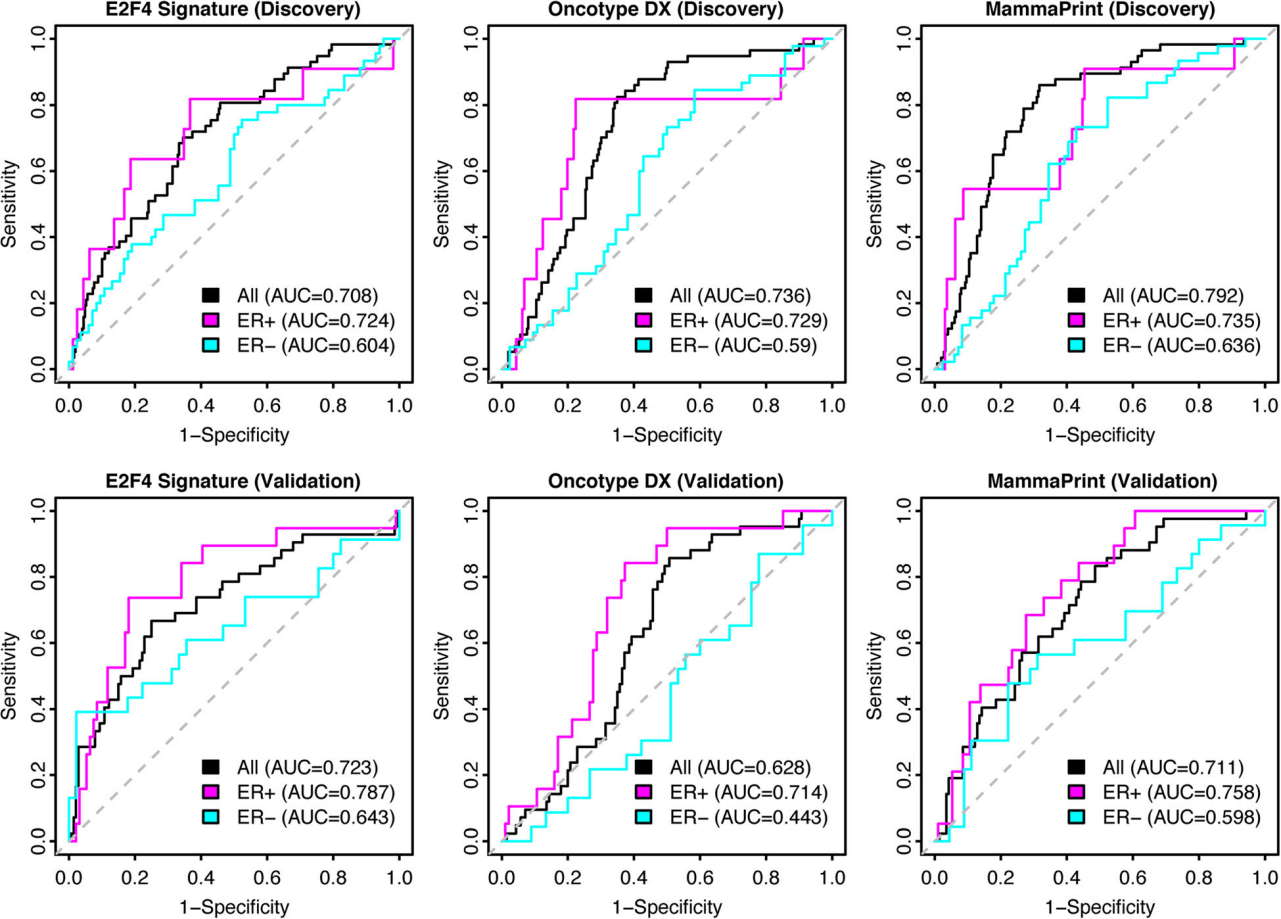
Comparison of pCR classification performance between the E2F4, Oncotype DX, and MammaPrint signatures in the Hatzis discovery and validation patient cohorts. pCR classification performance was evaluated using the E2F4, Oncotype DX, and Mammaprint signatures. ROC curves were plotted for all (*black*), ER+ (*magenta*) and ER- (*aqua*) patients. Grey dotted line indicates random classification performance

By using the E2F4 signature, we achieved good accuracy in classifying samples into pCR and RD. To benchmark our performance, we compared our results with the clinically-available prognostic assays Oncotype DX [9] and the MammaPrint 70-gene breast cancer recurrence assay [14]. To test the performance of each assay, we calculated the E2F4 iRAS, Oncotype DX score and MammaPrint 70-gene score on the Hatzis et al. discovery and validation cohorts individually and determined their accuracy by calculating the AUC, as we did previously (Fig. 3). Overall, the accuracy of the E2F4 signature was comparable to the other clinically-available assays in both the discovery and validation cohorts and this remained true when each assay was used to predict response in ER-positive and ER-negative patients.

Generally, when determining a patient’s treatment regimen, the results of these assays are combined with additional clinical information. To address this, we used a Random Forest classifier to determine how well our E2F4-based predictor performed in conjunction with clinical information and then compared the results to those using the MammaPrint and Oncotype DX signatures. Patients were first stratified into ER-positive and ER-negative groups and then for each group a classifier was trained using age, tumor stage, tumor grade, ER status, PR status, HER2 status, and lymph node metastasis status as features, in addition to scores from the E2F4, MammaPrint, or Oncotype DX signatures, depending on the comparison being made.

**Fig. 4:**
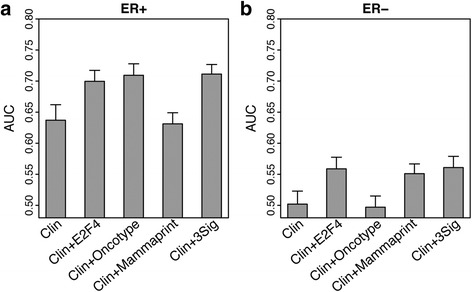
Classification performance after including clinicopathological features into pCR classification models. Comparison of AUCs between combinations of the E2F4 signature, Oncotype DX, MammaPrint, and clinicopathological features in **a** ER-positive patients and **b** ER-negative patients. Error bars indicate standard deviation calculated by performing 10-fold cross-validation 100 times

In ER-positive patients, integrating individual scores with clinical data improved the predictions from an AUC of 0.64 in clinical data only to 0.70 and 0.71 for the E2F4 and Oncotype DX scores, respectively (Fig. 4a). Interestingly, including the MammaPrint 70-gene signature did not improve predictive accuracy compared to clinical information alone. Using scores from all three signatures as features to predict pCR did not dramatically improve the AUC compared to either the E2F4 or Oncotype DX signatures alone, implying that combining the signatures together does not increase predictive value. In ER-negative patients, the average AUCs were much lower than those of the ER-positive patients. For this subtype, integrating the E2F4 and MammaPrint scores with clinical information led to a substantial boost in predictive accuracy, with AUCs rising from 0.50 to 0.56 and 0.55 in E2F4 and MammaPrint, respectively (Fig. 4b). As with the ER-positive cohort, including all three signatures as features along with clinical information did not result in a substantial improvement compared to the individual signatures, suggesting that combining these signatures provided little additional information. Based on these results, combining each of the gene signature scores with clinical information can improve the predictive accuracy compared to clinical information alone. Interestingly, the E2F4 signature was the only signature that added to predictive accuracy in both the ER-positive and ER-negative patient cohorts, suggesting that it may be a slightly more versatile test of neoadjuvant therapy response.

### Validation of the E2F4 signature in other datasets

**Fig. 5:**
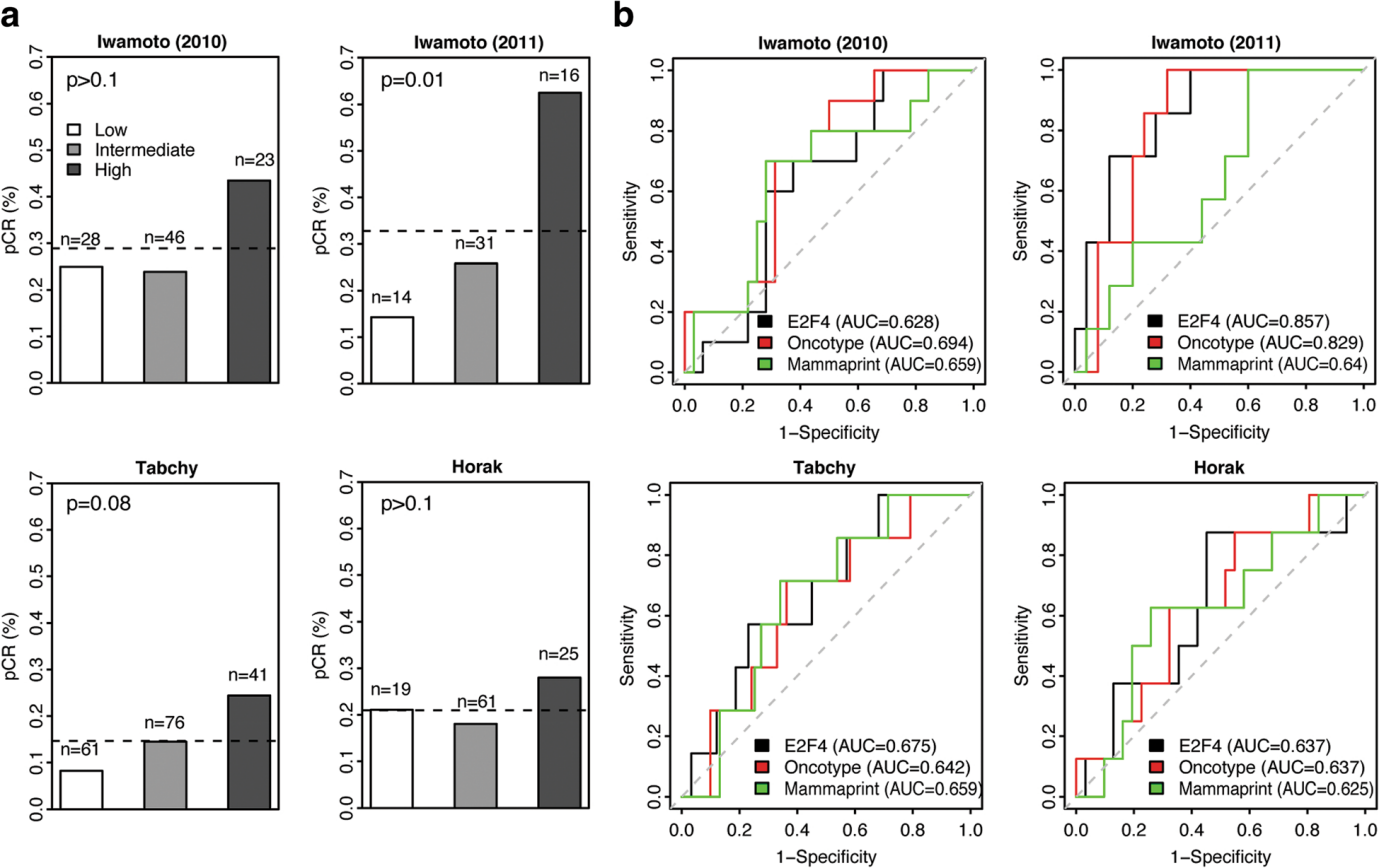
Comparison of pCR classification performance between the E2F4 signature, Oncotype DX, and MammaPrint in 4 independent datasets. **a** Percentage of pCR patients in low (*white*), intermediate (*grey*), and high (*dark grey*) E2F4 activity groups in the Iwamoto (2010), Iwamoto (2011), Tabchy, and Horak datasets. *P*-values were calculated using the χ^2^ test. **b** pCR classification performance using features from the E2F4 signature (black), Oncotype DX (*red*), and MammaPrint (*green*) in the Iwamoto (2010), Iwamoto (2011), Tabchy, and Horak datasets. Grey dotted line corresponds to random classification and an AUC of 0.5

To validate our results found from the Hatzis dataset, we applied our E2F4 signature to predict neoadjuvant response in four independent datasets by Iwamoto et al. (2010), Iwamoto et al. (2011) [26], Tabchy et al. [27], and Horak et al. [28]. For each dataset, we stratified patients into low, intermediate and high E2F4 groups and calculated the pCR rate among each as well as the AUCs to assess predictive accuracy for each of the 3 signatures: E2F4, OncotypeDX and MammaPrint. Across all 4 datasets, the pCR rate was highest in patients with high E2F4 activity (Fig. 5a). Patients with high E2F4 activity also had pCR rates far above the baseline pCR rate. These results were highly consistent with the Hatzis results, indicating that the E2F4 iRAS associations with pCR were not specific to a single dataset. In addition to these trends, the predictive accuracy of the E2F4 iRAS was consistent between datasets and performed comparably to the MammaPrint and Oncotype DX signatures (Fig. 5b). This reproducible performance further supports the E2F4 signature’s utility as a predictive test to determine the administration of neoadjuvant chemotherapy.

### A modified E2F4 signature composed of 33 genes is highly predictive of chemotherapy response.

**Fig. 6:**
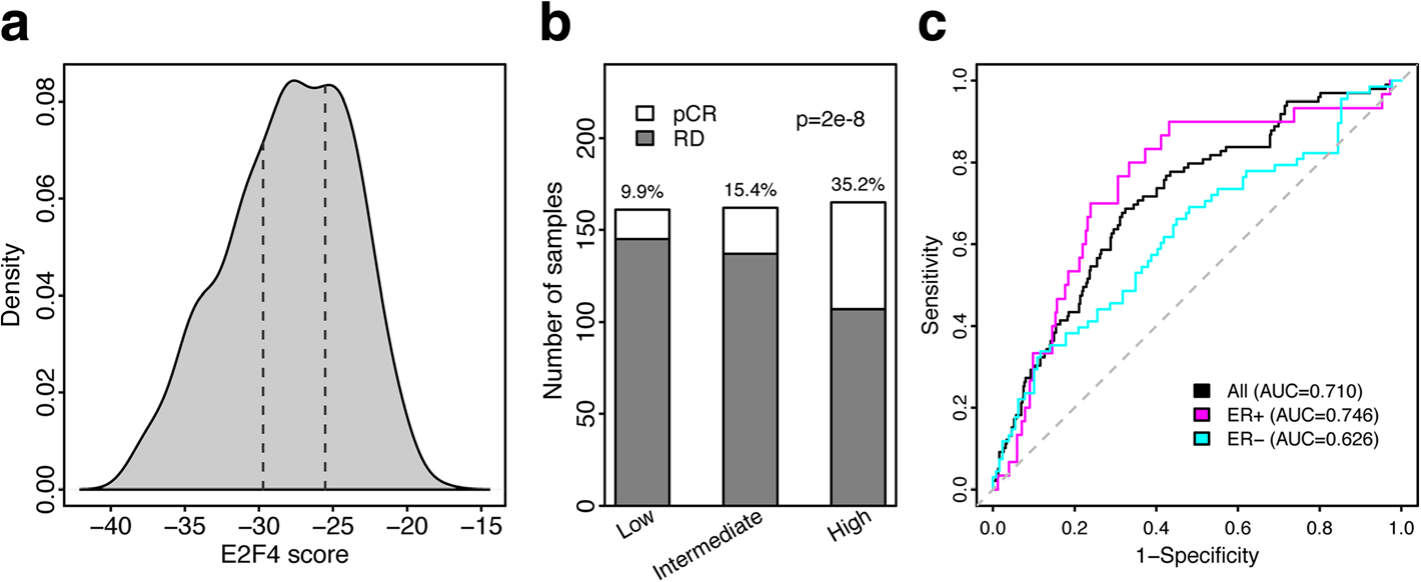
Performance of the modified E2F4 score when predicting pCR status in the Hatzis dataset. **a** Distribution of E2F4 activity scores based on the 33-gene signature. Vertical hashed lines indicate quantile divisions used to denote low (*white*), intermediate (*grey*), or high (*dark grey*) E2F4 activity. **b** Percentage of patients with pCR or RD that fall within the low (*white*), intermediate (*grey*) or high (*dark grey*) E2F4 score categories. *P*-value was calculated using the χ^2^ test. **c** ROC curves showing pCR classification performance when using the E2F4 scores calculated from the 33-gene signature in all (*black*), ER-positive (*magenta*), and ER-negative (*aqua*) patients. Grey dotted line corresponds to random classification and an AUC of 0.5

Calculation of E2F4 iRASs from the 199-gene signature requires a full patient microarray for normalization. While the iRASs from this signature proved to be predictive of neoadjuvant therapy response across datasets, the large amount of data required for calculation may be cost-prohibitive in a clinical setting. To address this, we reduced this signature down to a core set of 28 E2F4 target genes that best captured the information conferred by the original signature as well as 5 control genes used for normalization. Applying this signature to the Hatzis combined dataset, revealed a unimodal distribution of E2F4 iRASs as opposed to the bimodal distribution observed with the full signature. Thus, we sorted and equally divided patients into E2F4 low, intermediate, and high activity groups, as we could not use the two local maxima as cutoffs for class inclusion as we did with the full signature (Fig. 6a). We then calculated the number of patients in each category that achieved pCR and found, as with the full signature, that the rate of pCR increased moving from the low to intermediate to high classes (Fig. 6b). As a predictor of neoadjuvant chemotherapy response, the reduced signature’s performance proved to be comparable to that of the entire E2F4 signature (AUC = 0.710 versus 0.712 in the reduced and full signatures, respectively; (Fig. 6c). This trend was further observed when predicting ER-positive (AUC = 0.746 versus 0.712) and ER-negative patients (AUC = 0.626 versus 0.621). Together, these results suggest that the 33-gene E2F4 signature serves as an acceptable, more cost-effective substitute for the full signature in predicting neoadjuvant therapy response, making it a good candidate for clinical adaptation.

## Discussion

E2F4 is an essential cell cycle regulator that has been broadly implicated in tumorigenesis and cancer severity [29–31]. We previously developed a gene signature composed of E2F4 target genes predicted from ChIP-seq data and showed that this signature was a more effective tool to infer regulatory activity than expression of the transcription factor alone [17]. Patients with high E2F4 activity had significantly worse survival than patients with low activity, a trend consistent with other markers of tumor proliferation rate. In this study, we applied our signature to predict neoadjuvant therapy response and found that patients with high E2F4 iRASs were more likely to experience pCR than those with low and intermediate scores even when stratifying by breast cancer subtype. This result is unsurprising, as chemotherapeutic approaches target rapidly proliferating cells and high E2F4 regulatory activity is associated with high cellular proliferation rate [26, 32]. When stratifying patients into groups based on ER status, tumor stage, and molecular subtype, a high E2F4 iRAS continued to be indicative of improved pCR rate. The only time this trend did not hold was for the severe classes of breast cancer, defined by ER-negative status, high tumor stage, or a basal-like or HER2-enriched molecular subtype. We have shown previously that these subtypes exhibit high baseline E2F4 iRASs [17]. Thus, the E2F4 iRAS may not provide adequate resolution to identify the highly proliferative patients most likely to respond to neoadjuvant chemotherapy. Going forward, it will be important to improve our methods to better predict pCR rate for these severe subtypes of breast cancer.

The success of our E2F4-based predictions led us to assess the performance of our signature relative to the clinically available tests, Oncotype DX and MammaPrint. While these assays were originally intended to predict adjuvant chemotherapy response, recent reports have shown that they can also be applied to predict neoadjuvant chemotherapy. For example, the Oncotype DX recurrence score has been shown to predict response to neoadjuvant docetaxel, while the MammaPrint 70-gene signature was recently involved in studies predicting neoadjuvant chemotherapy response when combined with the Blueprint 80-gene molecular subtyping predictor [12, 13, 16, 33, 34]. As a univariate predictor, our E2F4 signature performed similarly to each clinical test, validating its use as a predictor of neoadjuvant therapy response. Additionally, when assessing the performance of each predictor in conjunction with clinical information, the E2F4 signature again performed comparably, and was the only signature to provide additional, albeit minor, information in both ER-positive and ER-negative sample cohorts. These findings indicate that the E2F4 signature may be able to provide predictive accuracy to a wider range of patients, though the utility of this extra information may be small.

The results from our E2F4 signature were promising, however calculation of the E2F4 iRAS requires the use of full patient microarrays, making it impractical for clinical use. To address this, we identified the E2F4 target genes most correlated with E2F4 iRAS and then combined these genes with a series of 5 control genes that could be used to calculate relative gene expression. The resulting 33-gene signature achieved similar predictive accuracy to the full signature, proving that this core set was adequate to infer E2F4 activity and predict neoadjuvant response. By distilling E2F4 activity into a reduced signature, we removed the microarray requirement for E2F4 iRAS calculation, resulting in a 33-gene panel that could instead be measured through more common clinical practices, such as RT-PCR. Going forward, this signature reduction method could easily be applied to additional microarray-dependent gene signatures, expediting their transition from the field of basic science to clinical application.

## Conclusion

In conclusion, we have demonstrated that a target gene-based signature of the transcription factor E2F4 can be used to predict response to neoadjuvant chemotherapy. Patients exhibiting high E2F4 scores were more likely to achieve pCR than patients with lower scores, further validating that the cellular proliferation rate in a patient’s tumor is a good biomarker for predicting neoadjuvant response. Our E2F4 signature performed comparably to signatures already available in the clinic, both as a univariate measurement and when integrated with clinical data. This performance was maintained when the signature was reduced from a microarray-dependent 199-gene signature to an independent 33-gene signature, indicating its potential for clinical adaptation. This study, while providing the basis for a potential clinical tool to predict neoadjuvant chemotherapy response, additionally serves as a paradigm for translating TF target gene-based signatures into predictive clinical tests, underscoring the importance of basic research in the clinical realm.

## Additional file

Additional file 1: Clinical characteristics by dataset of samples used in analysis. Sample size and clinical characteristics, including age, estrogen receptor status, neoadjuvant response status, and treatment protocol, for the samples used in each dataset involved in the study. (PDF 246 kb)

## Abbreviations

AUC: Area under the curve
BASE: Binding associated with sorted expression
DRFS: Distant recurrence free survival
ER: Estrogen receptor
GEO: Gene expression omnibus
iRAS: Individual regulatory activity score
pCR: Pathologic complete response
RCB: Residual cancer burden
RD: Residual disease
REACTIN: Regulatory activity inference
RT-PCR: Reverse-transcriptase polymerase chain reaction
