# Supplementary material for: The E2F4 prognostic signature predicts pathological response to neoadjuvant chemotherapy in breast cancer patients
Source: BMC Cancer. 2017 May 2;17:306. doi: 10.1186/s12885-017-3297-2 (PMC5414335; doi:10.1186/s12885-017-3297-2)
Supplement: Additional file 1: — Clinical characteristics by dataset of samples used in analysis. Sample size and clinical characteristics, including age, estrogen receptor status, neoadjuvant response status, and treatment protocol, for the samples used in each dataset involved in the study. (PDF 246 kb) [file 12885_2017_3297_MOESM1_ESM.pdf]

Additional File 1: Clinical characteristics by dataset of samples used in analysis

		Datasets N(%)						
		**Hatzis Discovery**	**Hatzis Validation**	**Hatzis Complete**	**Horak**	**Iwamoto (MDACC/IGR)**	**Iwamoto (USO-02103)**	**Tabchy**
**Stratum**		GSE25055	GSE25065	GSE25066	GSE41998	GSE22093	GSE23988	GSE20271
Total		310	198	508	279	103	61	178
Age in years	≤ 50	168 (54)	89 (45)	257 (51)	113 (41)	43 (44)	26 (43)	78 (44)
	> 50	142 (46)	109 (55)	251 (49)	166 (59)	54 (56)	35 (57)	100 (56)
	Mean (SD)	50 (10)	49 (11)	50 (10)	49 (11)	49 (11)	49 (9)	51 (11)
ER Status	ER positive	174 (56)	123 (62)	297 (58)	171 (61)	56 (54)	29 (48)	80 (45)
	ER negative	131 (42)	74 (37)	205 (40)	108 (39)	42 (41)	32 (52)	98 (55)
	Indeterminate	5 (2)	1 (1)	6 (1)	0 (0)	5 (5)	0 (0)	0 (0)
Neoadjuvant Response	pCR	57 (18)	42 (21)	99 (19)	40 (14)	28 (27)	20 (33)	26 (15)
	RD	249 (80)	140 (71)	389 (77)	230 (82)	69 (67)	41 (67)	152 (85)
	Unknown	4 (1)	16 (8)	20 (4)	9 (3)	6 (6)	0 (0)	0 (0)
Microarray Platform		Affymetrix HG-U133A	Affymetrix HG-U133A	Affymetrix HG-U133A	Affymetrix HG-U133A 2.0	Affymetrix HG-U133A	Affymetrix HG-U133A	Affymetrix HG-U133A
Treatment Protocol		Weekly paclitaxel x 12 or 3/weekly docetaxel x4 both followed by anthracycline x4. Endocrine therapy if ER+			1/ 3 weeks AC x 4 followed by either 1/3 weeks ixabepilone x4 or weekly paclitaxel x 12	4 course FAC	1/3 weeks FAC x 4 followed by weekly docetaxel	weekly paclitaxel × 12 followed by FAC × 4
